# Using Social Media to Disseminate Behavior Change Interventions: Scoping Review of Systematic Reviews

**DOI:** 10.2196/57370

**Published:** 2025-06-20

**Authors:** Porooshat Dadgostar, Qiuyuan Qin, Suiyue Cui, Laura Ellen Ashcraft, Reza Yousefi-Nooraie

**Affiliations:** 1 Department of Public Health Sciences University of Rochester Medical Center Rochester, NY United States; 2 Department of Biostatistics, Epidemiology, and Informatics Perelman School of Medicine University of Pennsylvania Philadelphia, PA United States

**Keywords:** social media, social networking, dissemination, behavioral interventions, health information

## Abstract

**Background:**

Compared with implementation, the conceptual frameworks, strategies, and outcomes of efforts to disseminate behavioral interventions are less developed. We conducted a scoping review of the systematic reviews of social media strategies to disseminate behavior change interventions. We focused on the common themes in the methodology and evaluation frameworks of social media–based dissemination strategies.

**Objective:**

This scoping review aims to identify common themes in the design, delivery, and impact assessment of social media–based dissemination strategies for behavior change interventions.

**Methods:**

We searched the Epistemonikos database (until 2024) to retrieve systematic reviews on social media dissemination. A total of 2 independent reviewers screened the abstracts and full texts. We extracted and classified the data on the characteristics of the included reviews and outcome assessments. We followed the reflexive thematic analysis steps to identify the main themes of the ingredients of the social media dissemination strategies.

**Results:**

We screened 613 records based on the title and abstract, followed by the assessment of 100 full texts of potentially eligible reviews. The 43 included reviews assessed a median of 20 empirical studies (IQ range 21). The study designs, intervention strategies, and evaluation measures of social media dissemination interventions were diverse. We classified the main themes of the ingredients of social media dissemination strategies into 4 main categories: 1-way spread (aiming for spread and diffusion, with little or no effort to develop 2-way communications or engage target users in conversation and feedback; n=37), invoking conversations (facilitating and enhancing the 1-way spread using conversational and community features of social media to promote dialogue among users or between the users and experts; n=21), peer motivation (facilitate sharing individual behavior on social media to receive confirmation, feedback, and support, to further personalize the dissemination; n=11), and miscellaneous (eg, dissemination through online multiplayer games; n=3). The main outcomes of dissemination efforts were reach and engagement (n=12), user perception of their knowledge, intention to change the behavior, feasibility and acceptability of the intervention (n=24), and impact on health and health-related behaviors (n=43). The majority of theoretical frameworks that were identified by the reviews were individual and social behavior change models (including the theory of planned behavior and Social Cognitive Theories). A smaller number of reviews also identified social and contextual models (eg, Social Network Theory), dissemination and implementation frameworks (eg, Diffusion of Innovation), and social marketing and action models (eg, community mobilization and Reader-to-Leader framework).

**Conclusions:**

Researchers use various features of social media (eg, peer-to-peer sharing, online engagement in conversations, one-on-one, or with a broad audience), formation of clusters and communities, and peer feedback to complement and enhance the 1-way dissemination. Further research is needed to inform the theoretical underpinnings and the interventional ingredients of social media dissemination strategies.

## Introduction

The definition of “dissemination” varies across studies. Compared with spontaneous and unplanned spread, dissemination is typically defined as the active and targeted distribution or transfer of new ideas, such as evidence-based interventions, to specific populations [1,2]. Identifying, reaching out to, and transferring innovations to potential users are crucial in the implementation of innovations [3,4]. Yet, the science of dissemination has been less explored than that of implementation, and little is known about the determinants, processes, and outcomes of dissemination efforts.

Dissemination strategies are actions and processes that promote and support the dissemination of information, conveying it from a source to the audience through the intentional development and selection of sources, content, and channels [1-3]. These actions may involve the development and tailoring of the content or message (eg, best practice advice and recommendations and policy briefs), identification and adaptation of channels (eg, in-person communication or social media platforms such as X Corp’s X and Meta’s Facebook, websites and weblogs, email, mass media, and podcasts), and engagement and activation of sources (eg, endorsement and distribution by valid, popular, target-specific individuals and organizations) [2,3]. Purposeful and systematic dissemination is essential for the uptake, spread, and use of health innovations, as the significant gap in translating research findings into practice and policy is partially attributed to ineffective and inadequate dissemination [3,5].

Social media is omnipresent as a potentially powerful dissemination platform, with more people accessing web-based content through social media links than through direct searches [6,7]. Thus, social media platforms play a prominent role in shaping and framing the public’s opinion at a low cost over short periods of time [6,8,9]. Leveraging the power of social media can help implementers overcome the barriers of scope and reach posed by traditional dissemination methods [10-13]. Social media also presents a new dimension to health care, as communication and collective actions through social interactions in online platforms have the power to influence health practices and policies [14,15].

However, little is known about the ingredients of social media–based dissemination strategies, the determinants of their success, and methods for assessing their effectiveness in disseminating behavior change interventions [6,16]. We conducted a scoping review that investigated the uses of social media in disseminating strategies in behavior change interventions. The goal of our review was to identify common themes in the design, delivery, and impact assessment of social media–based dissemination strategies on behavior change.

## Methods

### Overview

We conducted a scoping review of systematic reviews to understand the range, diversity, and characteristics of the literature, and identify potential research gaps [17]. Unlike systematic reviews, scoping reviews provide a broader conceptual overview of existing evidence, making them particularly useful for areas where the literature is dispersed across multiple disciplines or sources [18]. We followed the PRISMA-ScR (Preferred Reporting Items for Systematic Reviews and Meta-Analyses extension for Scoping Reviews) guidelines [19], as shown in Multimedia Appendix 1. We also adopted the methodological framework developed by Arksey and O’Malley [20], modified by Levac et al [21].

### Inclusion and Exclusion Criteria

We included systematic or scoping reviews that assessed the effect of social media–based strategies on the dissemination of health behavior change interventions.

### Systematic or Scoping Reviews

We included published systematic or scoping reviews in English that employed structured methods for literature searching, study selection, and synthesis. We excluded protocol papers, narrative reviews, and opinion pieces.

#### Social Media

We included studies that used social media as a part of their dissemination strategies. Following Carr and Hayes [22], we defined social media as “Internet-based channels that allow users to opportunistically interact and selectively self-present, either in real-time or asynchronously, with both broad and narrow audiences who derive value from user-generated content and the perception of interaction with others.”

#### Dissemination

We defined dissemination as an active and targeted process of identifying, reaching out to, and transferring knowledge to users [1,2].

#### Interventional Studies

We included reviews that assessed the effectiveness of dissemination strategies through empirical studies, in which researchers deliberately used social media to disseminate behavior change–related innovations or information. We excluded observational studies of social media user behavior patterns.

#### Behavior Change Interventions

We defined behavior change interventions as structured sets of actions designed to modify specific behavioral patterns. These patterns are typically assessed based on the prevalence or occurrence of certain behaviors within defined populations [23]. Popular examples include physical activity, smoking cessation, healthy sexual behavior, medication adherence, and adherence to guidelines.

#### Exclusion Criteria

We excluded non-English papers, unstructured (narrative) reviews, conference abstracts, unpublished reports, and research protocols.

#### Literature Search

We searched the Epistemonikos database to retrieve systematic reviews [24]. Epistemonikos is an open access meta database that screens 10 bibliographic databases on a daily or weekly basis (Cochrane Database of Systematic Reviews [CDSR], PubMed, Embase, Cumulative Index to Nursing & Allied Health [CINAHL], PsycINFO, Latin American and Caribbean Literature on Health Sciences [LILACS], Database of Abstracts of Reviews of Effects [DARE], The Campbell Collaboration Online Library, JBI Database of Systematic Reviews and Implementation Reports, and Evidence for Policy & Practice Information Centre [EPPI] Evidence Library) to identify systematic reviews relevant to health interventions [25]. As of June 2024, the database had retrieved 1,822,333 references from various databases and other sources, classified by human screeners and a machine learning algorithm, resulting in a total of 515,182 systematic reviews.

We used a sensitive search strategy to identify systematic and scoping reviews of dissemination studies, up to November 2024. The research team developed the search strategy using an iterative approach based on relevant keywords pertaining to social media and dissemination. We also reviewed the records obtained through Google Scholar search and complemented the keyword list of the Epistemonikos search strategy to improve the search recall. We limited the search to studies including the variations of the word “behavior” in the title and/or abstract. The comprehensive search strategy is provided in the Multimedia Appendix 2, which includes alternative terms related to social media and specific social media platforms, dissemination or spread, and behavior change.

#### Study Selection

We imported the retrieved records to the Rayyan platform [26]. Pairs of reviewers (PD, QQ, and SC) independently screened the abstracts, and disagreements were resolved through discussions with the third reviewer (RYN) in weekly meetings. We conducted a calibration review and, after reaching satisfactory agreements among reviewers, divided the included papers for full-text assessment. The team had regular meetings to reach a consensus on eligibility.

#### Data Charting and Thematic Analysis

A data-charting form was jointly developed to determine variables to extract, and was continuously revised and updated based on the new information identified through data extraction. Three reviewers (PD, QQ, and SC) reviewed each paper in pairs to identify the relevant components. The disagreements and confusions were resolved in regular meetings with RYN.

To identify the main themes of the ingredients of social media dissemination strategies, we needed to go beyond the description of strategies in the included reviews. We followed the principles of reflexive thematic analysis [27], following the recommendation by Arksey and O’Malley [20] and Colquhoun et al [28]. It involved familiarization with data, coding the intervention components, generating and reviewing themes, defining and naming themes, and writing up. The information on the ingredients of social media dissemination strategies was obtained from the full texts and or appendices of included reviews and, where needed, through the retrieval of the original empirical studies that were mentioned in the reviews.

## Results

### Background

**Figure 1:**
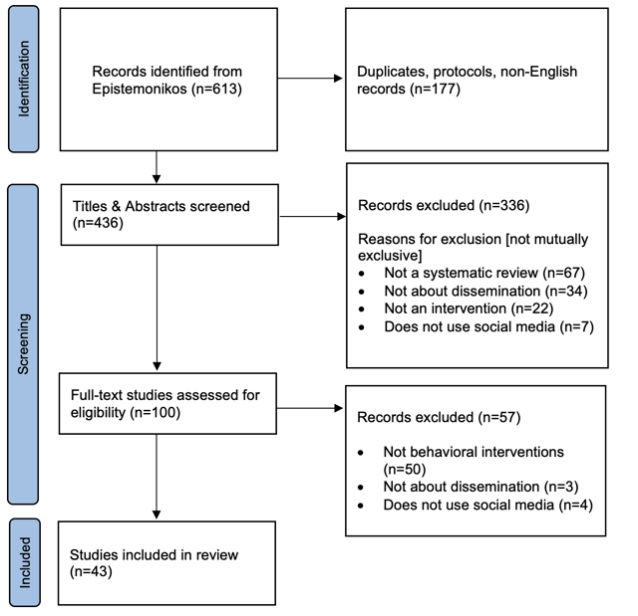
The PRISMA (Preferred Reporting Items for Systematic Reviews and Meta-Analyses) flow diagram illustrating the study selection procedure.

Figure 1 illustrates the PRISMA-ScR flowchart, outlining the process of study selection. The Epistemonikos search in November 2024 yielded 613 potential systematic reviews, of which 513 were excluded through title and abstract screening. After reviewing the full texts of the reviews for eligibility, we excluded an additional 55 records. We included and extracted the data from 43 reviews. The number of interventional studies included in these reviews varied from 7 to 143, with a median of 20 (IQR 21). Thirty-one out of 43 (72%) reviews were published in or after 2020 [16,29-58]. Out of 43, 17 were from the United States [29,34,37,39-41,45,49,52,57-64], 10 from Australia [36,47,48,50,54,56,65-68] 6 from the United Kingdom [31,32,38,44,51,69], 3 from Canada [16,42,70], and 7 from the rest of the world [35,46], Italy [33], Japan [43], Netherlands [30], Saudi Arabia [55], and Singapore [53]. The most frequent behaviors targeted were physical activity and healthy eating, followed by smoking cessation and vaccination, and testing. Twenty-eight studies reported a risk of bias assessment or commented on the quality of primary studies [32-34,36,38,40,41,43,44,47-53,55,56,58-60,63-68,70].

### Target Populations

As shown in the Multimedia Appendix 3, the included systematic reviews summarized the findings of reviews that focused on the following categories.

#### General Population

A total of 15 reviews focused on the general population with little specification of demographics [29,35,36,40,43-45,47,49, 55,58,63,64,68,69]. Of the reviews that specified the target population, 2 focused on adults [16,48]; one on children aged between 5 and 12 years [32]; 9 on young adults and adolescents [38,42,46,54,60-62,66,67]; 2 on rural communities and people with low socioeconomic status [30,37]; 2 on expectant mothers or mothers with infants [51,57]; 2 on young women [50,56]; and one on university students [71].

#### Patients

A total of 2 reviews focused on specific clinical conditions: obesity and metabolic syndrome [59] and clinical indication of mental illness [65].

#### Multiple Groups

A total of 7 reviews included studies targeting multiple groups: one review included studies on clinicians, health workers, medical students, and patients [31]; one on healthy adults, overweight or adults living with obesity, chronically ill patients, pregnant mothers, and children of different age groups [53]; one on adolescents and their parents [34]; one on unvaccinated youth and their parents [52]; one on different groups like men who have sex with men, people living with HIV, adolescents, and college students [39]; one on overweight or healthy sedentary individuals [70], one on Black and Latinx men who have sex with men, and women younger than 29 years [41].

#### Study Designs

All reviews except one [58] reported the inclusion of randomized controlled trials (RCTs). The quasi-experimental study design was also commonly reported among the included reviews [33,47,50,52,54,56,61,62,67]. Fifteen reviews included studies with pre-post designs [16,29,33,34,37,39,42,46,48,49, 59,60,62,63,69]. Observational studies were included in 7 reviews [37,39,41,51,55,58,68]. A total of 3 reviews included qualitative studies [37,38,57], and 5 included mixed methods studies [37,38,42,52,57]. One review did not report the study design of their included studies [44].

#### The Ingredients of the Social Media Dissemination Strategies

We generated a classification for each included article by thematically categorizing the reported dissemination strategies into the following groups: 1-way spread, invoking conversations, peer motivation, and miscellaneous. A total of 3 reviews did not provide sufficient information about the components of interventions used in their included studies [30,33,53]. One review did not report any included studies with a focus on dissemination through social media, despite an appropriate eligibility criterion [65]. We summarized these categories in the Multimedia Appendix 3.

#### One-Way Spread

One-way spread involves posting content on social media platforms (such as X [formerly Twitter], Facebook, Instagram, LinkedIn, blogs, and webcasting), aiming for dissemination, with minimal effort to develop 2-way communication or engage target users in conversation and feedback. Thirty-seven reviews included interventions that incorporated 1-way spread of dissemination strategies [16,29,31,32,34-45,47-52,54,55, 57-64,66-70]. Examples include posting evidence-based messages on public social media platforms (eg, X [formerly Twitter]) or specialized groups (eg, Facebook groups for patients with similar conditions) and training opinion leaders to post on social media.

#### Invoking Conversations

The reviews identified several studies that included interventions aimed at initiating or facilitating conversation and dialogue among users and experts, thereby promoting and enhancing dissemination. The communications may aim to provide clarifications and personalized instructions to facilitate the adoption of behavior and its dissemination through trusted sources (eg, opinion leaders or friends) [16]. This category can be further broken down into creating communities for peer discussions and promoting private communications with experts or among select individuals.

Researchers created specialized communities to promote conversations among peers, such as closed social media groups only accessible to the target users (eg, patients or individuals committed to quitting smoking). Twenty-one reviews indicated the development of and posting materials in closed communities, like discussion boards and forums, as the dissemination method [32,37-39,42,44-49,51,52,54,55,61,64,67-69,72]. Alternatively, researchers used social media platforms to facilitate one-to-one communication. Two of these systematic reviews included conversations with experts [37,70]. A total of 15 systematic reviews included studies that invoked private communications among target users, such as peer mentorship and sharing information in private chats [37,39,42,45-48,51,54,57,60,64, 67-69].

#### Peer Motivation

Dissemination strategies in this category aimed to facilitate sharing individual behavior on social media to receive confirmation, feedback, and support from others. This category aimed to reinforce one-way dissemination through peer influence and personalization.

This category primarily focuses on posting progress updates on social media. Eleven reviews indicated that users promoted the intervention by posting their personal improvements on social media, either manually or automatically through apps [16,33,37,41,46,51,54,63,64,69,70]. Examples include sharing dietary or tobacco abstinence progress with a peer group or publicly on social media, where one can receive compliments and support from others.

#### Miscellaneous

A total of 3 reviews included studies that developed socially interactive social media–based games that involved inviting friends and sharing progress with them, interaction with peers, and the communication of tailored feedback to reinforce dissemination [34,46,64]. Martin et al [46] also included studies that used social media dissemination through fictional characters with social media pages that promoted healthy behaviors. Table 1 shows the summary of the ingredients of social media dissemination strategies in the included studies, sorted by the ingredient type.

**Table 1:** The ingredients of social media dissemination strategies in the included studies.

Study	1-way spread	Invoking conversations	Peer motivation	Miscellaneous
Elaheebocus et al [69]	✓	✓	✓	
Eppes et al [37]	✓	✓	✓	
McKeon eta al [47]	✓	✓	✓	
Naslund et al [64]	✓	✓	✓	
Sewak et al [54]	✓	✓	✓	
Williams et al [70]	✓	✓	✓	
Choi et al [34]	✓	✓		✓
Buja et al [33]	✓		✓	
Kudrati et al [41]	✓		✓	
Laranjo et al [63]	✓		✓	
Orchard and Nicholls [51]	✓		✓	
Bhatt et al [31]	✓	✓		
Brigden et al [32]	✓	✓		
Chau et al [60]	✓	✓		
Draganidis et al [36]	✓	✓		
Goodyear et al [38]	✓	✓		
Guse et al [61]	✓	✓		
Hsu et al [67]	✓	✓		
Ibrahim et al [39]	✓	✓		
Kulandaivelu et al [42]	✓	✓		
Li et al [44]	✓	✓		
Limaye et al [45]	✓	✓		
Maher et al [68]	✓	✓		
Mersha et al [48]	✓	✓		
Niu et al [49]	✓	✓		
Ou et al [52]	✓	✓		
Talie et al [55]	✓	✓		
Wu et al [57]	✓	✓		
Acuna et al [29]	✓			
An et al [59]	✓			
Carson et al [66]	✓			
de Oliveira Júnior et al [35]	✓			
Jones et al [62]	✓			
Kim et al [40]	✓			
Kuwahara et al [43]	✓			
O'Connor et al [50]	✓			
Yeh et al [58]	✓			
Martin et al [46]		✓	✓	
Simeon et al [16]			✓	
Brijnath et al [65]				✓
Al-Dhahir et al [30]	Not enough information provided	Not enough information provided	Not enough information provided	Not enough information provided
Seid et al [53]	Not enough information provided	Not enough information provided	Not enough information provided	Not enough information provided
Watson-Mackie et al [56]	Not enough information provided	Not enough information provided	Not enough information provided	Not enough information provided

#### Outcome Assessment

We classified the primary reported outcomes into 4 categories by building on previous work by Baumann et al [1]. As demonstrated in the table in the Multimedia Appendix 3, we classified the primary reported outcomes into 4 categories.

#### Diffusion and Engagement

A total of 12 reviews included studies that reported users’ engagement with social media platforms or apps as diffusion and engagement indicators, including social media impressions, shares, tweets, retweets, likes, comments, frequency of interactions, participation in peer communications, survey participation, downloads, and other bibliometric measures [31,36-38,44,45,50-52,55,60,64].

#### Users’ Perceptions

Around 7 reviews included studies that assessed users’ perception of the feasibility, usefulness, and acceptability of the interventions through self-report [30,31,46,47,64-66]. A total of 17 reviews included studies that used tests, screening, and questionnaires to assess user perception, attitude, and knowledge [33,35,36,39,41,44,49,51,52,54,55,57,58,61,62,66, 67].

#### Impact on Health and Health-Related Behaviors

All included reviews reported the effect of social media interventions on health and clinical outcomes. This included self-reported healthy behavior, indicators of adherence to the desired intervention, measures of psychological well-being and quality of life, and indicators of improvement in health-related outcomes (such as weight management or smoking cessation).

#### Theoretical Frameworks and Models

Many theories and conceptual frameworks have been reported in the included reviews, including individual-level behavior change theories (eg, Theory of Planned Behavior, Health Belief Model, self-efficacy, and cognitive behavioral approaches) [30,34,36,37,44,47,58,60,61,68,69], stage-based and motivational theories (eg, Trans-theoretical Model, Self-Determination Theory, and Technology Acceptance Model) [34,36,37,47,56,60,61,69,73], socially focused behavioral theories (eg, Social Learning Theory and Social Cognitive Theory) [30,34,37,41,42,44,46,47,56,58-61,63,66-69], broader social and contextual models (eg, Social Network Theory, social norms theories, and Social Identity Theory) [42,58,60,63,69], dissemination and implementation of theoretical models and frameworks (eg, Diffusion of Innovations, and Theoretical Domains Framework) [34], and marketing and community-focused models (eg, community mobilization, Reader-to-Leader framework, bystander education, and social marketing) [42,58]. Because many of these theories and models span multiple categories, this classification necessarily simplifies their nuanced conceptual overlaps.

## Discussion

### Principal Findings

This scoping review aimed to provide a typology of the ingredients and characteristics of social media dissemination strategies for disseminating behavioral interventions. We included 43 reviews, mainly targeting the general population. The 1-way spread of content on social media was the primary strategy. This was complemented and enhanced by more communicative strategies, relying on social relationship facilitated through social media platforms, to invoke small or large group conversations among peers, with experts, or with influential actors (ie, opinion leaders, and to facilitate the sharing of personal behavior and progress to raise attention of and receive positive feedback from peers. These communicative activities would enhance 1-way spread through incorporating interpersonal trust, collective norms, and individualized feedback into the messages. Few studies also used collaborative projects to create and disseminate knowledge collectively [74]. The impact of interventions was typically measured through health and clinical outcomes, as well as individual perceptions, with less emphasis on indicators of dissemination and diffusion processes.

### Dissemination Strategy Ingredients

The included strategies were often multi-component and complex. Other investigators have observed the complexity of social media strategies, which makes it difficult to assess their effectiveness and the mechanisms of impact [75,76]. We identified the lack of clarity in definitions as a critical factor contributing to this complexity, as it was often unclear what the authors intended by “social media” and “dissemination.” Informed by structured definitions of social media [22,77], we specified the following characteristics for a platform to be considered a social media platform: internet-based, providing opportunities for users to interact and self-present, in real-time or asynchronously, and either privately or publicly, in small groups or on a broad scale. Similarly, there were inconsistencies in the operationalization of the concept of dissemination. We defined dissemination as the targeted process of identifying and reaching out to users for engagement. According to this definition, many social media strategies would be considered dissemination efforts, even though terms like “dissemination,” “diffusion,” or “spread” were not mentioned in some of the included reviews.

Unlike traditional means of communication, social media offers unique opportunities for bidirectional interactions through verbal (comments) and nonverbal (liked and shared posts, followed accounts, and the use of emojis and picture-based media) communication. These communication features engage the target users with the message in a more profound way, transforming the messages to provide opportunities for user input and customization, and ultimately converting them into personal stories that enhance adaptation and adoption [73]. In other words, through 2-way communication, users are also engaged in recreating messages by adding their interpretations and feedback [78]. Through this process, the original messages are adapted to the needs and unique experiences of different user groups; however, the content may evolve in ways not intended by the original developers. Newer models of communication, such as the Dynamic Transactional Model, recognize the 2-way and dynamic nature of content creation and within and between subject transactions that happen through social media conversations [79].

Providing opportunities for peer motivation also enhances user engagement through other cognitive processes, as users observe the feasibility of behavior change in their peers’ progress. This would influence “perceived behavioral control” and “subjective norms” as 2 key determinants of behavior change [80]. People who share their progress toward the new behavior are acting as new sources of messages, influencing not only their peers’ motivation and behavior but also being influenced by their peers as they receive positive feedback and compliments. This will enhance the feedback loops of reinforcement.

Intervention developers should consider various factors when developing social media strategies to disseminate behavioral health messages, including the content (eg, relevance to the audience, evidence base), the channels (eg, technological limitations, privacy, and access), the audience (eg, literacy skills, knowledge and attitude, incentives, and information overload), and broader contextual barriers (eg, norms and misinformation) [5]. In addition, they should recognize the possibility of the evolution of the messages and the role of the audience as the new cocreators.

Social media interventions built upon the one-way spread of content require platforms that optimize reach and visibility for intended users. Interventions designed to invoke conversations necessitate platforms that facilitate dialogue, reflection, and information exchange among users and between users and experts. On the other hand, interventions focusing on peer motivation should develop indicators of progress that are both meaningful and easy to share, facilitating other users to provide positive feedback and compliments.

One-way spread requires developing messages in a format that is eye-catching and accessible, making them easy to consume. In conversational interventions, the messages become digested and reshaped as users engage with and discuss them. In the latter interventions, researchers have less control over the final content and format, instead using social media platforms to contextualize and adapt interventions for different user groups. Peer motivation interventions rely on social influence and peer pressure to engage new potential users with the intervention.

### Theoretical Underpinnings and Outcomes

Our findings suggest that, although many studies have reported theories and frameworks that informed interventional studies of social media dissemination, they primarily relied on classical theories of individual behavior change. The use of theories related to dissemination, communication, and community action was infrequent. This is a frequent limitation in dissemination research [1,81,82]. Theoretical frameworks offer researchers a systematic approach to plan, organize, evaluate, and explain the factors that influence outcomes [83]. Failure to inform the study with sound and relevant theoretical models limits the study's usefulness, as it is unclear what is meant by dissemination, how its related constructs are measured, and how different intervention components correspond to dissemination processes [1,81].

In 2022, Baumann et al [1] presented a taxonomy of dissemination frameworks in a scoping review of the literature. Among the theories and frameworks that they identified in their review, only the Diffusion of Innovation (one included review), the Technology Acceptance Model (one included review), and the Theory of Planned Behavior (9 included reviews) were mentioned in our studies. Several other relevant theoretical models from our included reviews were not mentioned in Baumann’s assessment. Examples include theories that pertain to the role of social dynamics on individual behavior (eg, Social Learning Theory and Social Cognitive Theory), as well as marketing and community-focused models (eg, community mobilization, Reader-to-Leader framework, and bystander education), which are particularly relevant to social media behavior change. For example, models of social mobilization and community action aim to explain the processes and interventions to facilitate the collective behavior of communities to address shared concerns [84], and have been applied to social media and health promotion studies [11,85]. Social marketing approaches also provide helpful strategies for more effective use of social media for health promotion [86,87], which inherently involve dissemination activities.

Similarly, we found that most dissemination studies have predominantly focused on health behavior change and clinical outcomes, with less emphasis placed on dissemination process indicators, such as reception, awareness, persuasion, and emotional reactions of users, which may ultimately contribute to behavior change and improvement in clinical outcomes.

### Limitations

There are several limitations to this scoping review. First, we relied on the digested data from the included systematic reviews and, with some exceptions, did not extract data from the original empirical studies they included. In addition, we did not incorporate the potential overlap among those empirical studies across the included reviews. Consequently, some empirical findings may have been counted multiple times, potentially inflating the apparent strength or consistency of evidence for certain dissemination strategies. However, this limitation would not impact the nature of the themes of the ingredients of dissemination strategies that were identified in this study. We also only included reviews published in English-language peer-reviewed journals, which may have led to the exclusion of relevant data from other potentially relevant literature syntheses. For practical reasons, we limited our search to reviews that included the variations of the term “behavior” in the title and abstract, which might have led to missing papers that focused on specific types of behavior, such as vaccine uptake, without mentioning the term. Also, systematic reviews included in this study were identified from a single database, though it includes the records from 10 other popular bibliographic databases and is updated frequently. Given the rapidly evolving nature of dissemination practices and the inherent time lag involved in conducting and publishing systematic reviews, there is a risk that more recent, innovative, or emerging dissemination strategies were not captured in the included reviews.

In addition, we did not assess the methodological quality of the included reviews or their original studies. This is consistent with the goals of this scoping review, as we aimed to demonstrate the breadth of approaches and techniques used to develop social media dissemination strategies and ways to assess their success. Hence, we did not intend to summarize the effectiveness of these interventions.

Furthermore, this review was not preregistered, which could impact its transparency and reproducibility. However, given the inductive and evolving nature of our analysis, we maintained an internal audit trail of modifications and employed an open and communicative approach within the review team to address emerging issues.

### Conclusion

Social media dissemination strategies for behavioral interventions often focus on the 1-way spread of messages to the intended audience, using social media platforms as mass media channels. Investigators enhance and expand the 1-way spread, using the communicational features of social media. These strategies include activities that facilitate conversations among peers (eg, discussion forums and groups) and between users and experts (through private chats and closed communities), as well as promoting peer motivation through peer feedback and confirmation. These communicational enhancements personalize the disseminated health messages and enhance their adoption through interpersonal trust, while dynamically changing the nature of the messages as they spread through and are recreated by the users.

We propose that intervention developers design social media dissemination strategies based on the scope of the target audience, the extent to which they intend to engage the audience as cocreators and influencers, and the intended role of experts in the dissemination process. The resulting theoretically informed interventions will better resonate with the target audience and encourage active participation in the dissemination. Although we are currently unable to assess the strength of the evidence supporting the 3 categories of social media dissemination strategies, this could provide valuable direction for future investigations.

Future research could further explore the ingredients of social media dissemination strategies, interactions among interventional components, and their impact on dissemination and behavioral outcomes by synthesizing quantitative measures of impact and effectiveness across studies.

## Appendix

Multimedia Appendix 1 PRISMA-ScR checklist.

Multimedia Appendix 2 Search Strategy.

Multimedia Appendix 3 The ingredients of social media dissemination strategies in the included studies.

## Abbreviations

CDSR: Cochrane Database of Systematic Reviews
CINAHL: Cumulative Index to Nursing & Allied Health
DARE: Database of Abstracts of Reviews of Effects
EPPI: Evidence for Policy & Practice Information Centre
LILACS: Latin American and Caribbean Literature on Health Sciences
PRISMA-ScR: The Preferred Reporting Items for Systematic Reviews and Meta-Analyses extension for Scoping Reviews
RCT: randomized controlled trial

## Data Availability

All data generated or analyzed during this study are included in this published article (and its supplementary information files).

## References

[1] Baumann AA, Hooley C, Kryzer E, Morshed AB, Gutner CA, Malone S, Walsh-Bailey C, Pilar M, Sandler B, Tabak RG, Mazzucca S (2022). A scoping review of frameworks in empirical studies and a review of dissemination frameworks. Implement Sci.

[2] Weiner BJ, Lewis CC, Sherr KH (2023). Practical Implementation Science: Moving Evidence Into Action.

[3] Brownson RC, Eyler AA, Harris JK, Moore JB, Tabak RG (2018). Getting the word out: New approaches for disseminating public health science. J Public Health Manag Pract.

[4] Rogers EM (2010). Diffusion of Innovations, 4th Edition.

[5] Chapman E, Haby MM, Toma TS, de Bortoli MC, Illanes E, Oliveros MJ, Barreto JOM (2020). Knowledge translation strategies for dissemination with a focus on healthcare recipients: an overview of systematic reviews. Implement Sci.

[6] Gough A, Hunter RF, Ajao O, Jurek A, McKeown G, Hong J, Barrett E, Ferguson M, McElwee G, McCarthy M, Kee F (2017). Tweet for behavior change: Using social media for the dissemination of public health messages. JMIR Public Health Surveill.

[7] Seiler J, Libby TE, Jackson E, Lingappa JR, Evans WD (2022). Social media-based interventions for health behavior change in low- and middle-income countries: systematic review. J Med Internet Res.

[8] Chew C, Eysenbach G (2010). Pandemics in the age of Twitter: content analysis of Tweets during the 2009 H1N1 outbreak. PLoS One.

[9] Corley CD, Cook DJ, Mikler AR, Singh KP (2010). Text and structural data mining of influenza mentions in web and social media. Int J Environ Res Public Health.

[10] Castillo LIR, Hadjistavropoulos T, Brachaniec M (2021). The effectiveness of social media in the dissemination of knowledge about pain in dementia. Pain Med.

[11] Chen J, Wang Y (2021). Social media use for health purposes: systematic review. J Med Internet Res.

[12] Stellefson M, Paige SR, Chaney BH, Chaney JD (2020). Evolving role of social media in health promotion: Updated responsibilities for health education specialists. Int J Environ Res Public Health.

[13] Petkovic J, Duench S, Trawin J, Dewidar O, Pardo Pardo J, Simeon R, DesMeules M, Gagnon D, Hatcher Roberts J, Hossain A, Pottie K, Rader T, Tugwell P, Yoganathan M, Presseau J, Welch V (2021). Behavioural interventions delivered through interactive social media for health behaviour change, health outcomes, and health equity in the adult population. Cochrane Database Syst Rev.

[14] Bou-Karroum L, El-Jardali F, Hemadi N, Faraj Y, Ojha U, Shahrour M, Darzi A, Ali M, Doumit C, Langlois EV, Melki J, AbouHaidar GH, Akl EA (2017). Using media to impact health policy-making: an integrative systematic review. Implement Sci.

[15] Moorhead SA, Hazlett DE, Harrison L, Carroll JK, Irwin A, Hoving C (2013). A new dimension of health care: systematic review of the uses, benefits, and limitations of social media for health communication. J Med Internet Res.

[16] Simeon R, Dewidar O, Trawin J, Duench S, Manson H, Pardo Pardo J, Petkovic J, Hatcher Roberts J, Tugwell P, Yoganathan M, Presseau J, Welch V (2020). Behavior change techniques included in reports of social media interventions for promoting health behaviors in adults: content analysis within a systematic review. J Med Internet Res.

[17] Hunt H, Pollock A, Campbell P, Estcourt L, Brunton G (2018). An introduction to overviews of reviews: planning a relevant research question and objective for an overview. Syst Rev.

[18] Peters MDJ, Godfrey CM, Khalil H, McInerney P, Parker D, Soares CB (2015). Guidance for conducting systematic scoping reviews. Int J Evid Based Healthc.

[19] Tricco AC, Lillie E, Zarin W, O'Brien KK, Colquhoun H, Levac D, Moher D, Peters MDJ, Horsley T, Weeks L, Hempel S, Akl EA, Chang C, McGowan J, Stewart L, Hartling L, Aldcroft A, Wilson MG, Garritty C, Lewin S, Godfrey CM, Macdonald MT, Langlois EV, Soares-Weiser K, Moriarty J, Clifford T, Tunçalp Ö, Straus SE (2018). PRISMA extension for scoping reviews (PRISMA-ScR): checklist and explanation. Ann Intern Med.

[20] Arksey H, O'Malley L (2005). Scoping studies: towards a methodological framework. Int J Soc Res Methodol.

[21] Levac D, Colquhoun H, O'Brien KK (2010). Scoping studies: advancing the methodology. Implement Sci.

[22] Carr CT, Hayes RA (2015). Social media:defining, developing, and divining. Atl J Commun.

[23] Michie S, van Stralen MM, West R (2011). The behaviour change wheel: a new method for characterising and designing behaviour change interventions. Implement Sci.

[24] Rada G, Pérez D, Araya-Quintanilla F, Ávila C, Bravo-Soto G, Bravo-Jeria R, Cánepa A, Capurro D, Castro-Gutiérrez V, Contreras V, Edwards J, Faúndez J, Garrido D, Jiménez M, Llovet V, Lobos D, Madrid F, Morel-Marambio M, Mendoza A, Neumann I, Ortiz-Muñoz L, Peña J, Pérez M, Pesce F, Rain C, Rivera S, Sepúlveda J, Soto M, Valverde F, Vásquez J, Verdugo-Paiva F, Vergara C, Zavala C, Zilleruelo-Ramos R, Epistemonikos project (2020). Epistemonikos: a comprehensive database of systematic reviews for health decision-making. BMC Med Res Methodol.

[25] Epistemonikos database methods. Epistemonikos.

[26] Ouzzani M, Hammady H, Fedorowicz Z, Elmagarmid A (2016). Rayyan-a web and mobile app for systematic reviews. Syst Rev.

[27] Braun V, Clarke V, Hayfield N (2023). Thematic analysis: A reflexive approach. Qualitative Psychology: A Practical Guide to Research Methods. 4th ed:.

[28] Colquhoun HL, Levac D, O'Brien KK, Straus S, Tricco AC, Perrier L, Kastner M, Moher D (2014). Scoping reviews: time for clarity in definition, methods, and reporting. J Clin Epidemiol.

[29] Acuna N, Vento I, Alzate-Duque L, Valera P (2020). Harnessing digital videos to promote cancer prevention and education: a systematic review of the literature from 2013-2018. J Cancer Educ.

[30] Al-Dhahir I, Reijnders T, Faber JS, van den Berg-Emons RJ, Janssen VR, Kraaijenhagen RA, Visch VT, Chavannes NH, Evers AWM (2022). The barriers and facilitators of eHealth-based lifestyle intervention programs for people with a low socioeconomic status: Scoping review. J Med Internet Res.

[31] Bhatt NR, Czarniecki SW, Borgmann H, van Oort IM, Esperto F, Pradere B, van Gurp M, Bloemberg J, Darraugh J, Rouprêt M, Loeb S, N'Dow J, Ribal MJ, Giannarini G, EAU Guidelines Office Dissemination Committee (2021). A systematic review of the use of social media for dissemination of clinical practice guidelines. Eur Urol Focus.

[32] Brigden A, Anderson E, Linney C, Morris R, Parslow R, Serafimova T, Smith L, Briggs E, Loades M, Crawley E (2020). Digital behavior change interventions for younger children with chronic health conditions: Systematic review. J Med Internet Res.

[33] Buja A, Lo Bue R, Mariotti F, Miatton A, Zampieri C, Leone G (2024). Promotion of physical activity among university students with social media or text messaging: a systematic review. Inquiry.

[34] Choi J, Tamí-Maury I, Cuccaro P, Kim S, Markham C (2023). Digital health interventions to improve adolescent HPV vaccination: a systematic review. Vaccines (Basel).

[35] de Oliveira Júnior AJ, Oliveira JM, Bretz YP, Mialhe FL (2023). Online social networks for prevention and promotion of oral health: a systematic review. Can J Dent Hyg.

[36] Draganidis A, Fernando AN, West ML, Sharp G (2024). Social media delivered mental health campaigns and public service announcements: a systematic literature review of public engagement and help-seeking behaviours. Soc Sci Med.

[37] Eppes EV, Augustyn M, Gross SM, Vernon P, Caulfield LE, Paige DM (2023). Engagement with and acceptability of digital media platforms for use in improving health behaviors among vulnerable families: systematic review. J Med Internet Res.

[38] Goodyear VA, Wood G, Skinner B, Thompson JL (2021). The effect of social media interventions on physical activity and dietary behaviours in young people and adults: a systematic review. Int J Behav Nutr Phys Act.

[39] Ibrahim K, Kahle EM, Christiani Y, Suryani S (2024). Utilization of social media for the prevention and control of HIV/AIDS: a scoping review. J Multidiscip Healthc.

[40] Kim TV, Pham TND, Phan P, Le MHN, Le Q, Nguyen PT, Nguyen HT, Nguyen DX, Trang B, Cao C, Gurakar A, Hoffmann CJ, Dao DY (2024). Effectiveness and implementation of decentralized, community- and primary care-based strategies in promoting hepatitis B testing uptake: a systematic review and meta-analysis. EClinicalMedicine.

[41] Kudrati SZ, Hayashi K, Taggart T (2021). AIDS Behav.

[42] Kulandaivelu Y, Hamilton J, Banerjee A, Gruzd A, Patel B, Stinson J (2023). Social media interventions for nutrition education among adolescents: scoping review. JMIR Pediatr Parent.

[43] Kuwahara K, Sakamoto M, Ishizuka R, Kato M, Akiyama M, Ishikawa H, Kiyohara K (2023). Effect of digital messages from health professionals on COVID-19-related outcomes: a systematic review of randomized controlled trials. J Infect Public Health.

[44] Li L, Wood CE, Kostkova P (2022). Vaccine hesitancy and behavior change theory-based social media interventions: a systematic review. Transl Behav Med.

[45] Limaye RJ, Holroyd TA, Blunt M, Jamison AF, Sauer M, Weeks R, Wahl B, Christenson K, Smith C, Minchin J, Gellin B (2021). Social media strategies to affect vaccine acceptance: a systematic literature review. Expert Rev Vaccines.

[46] Martin P, Cousin L, Gottot S, Bourmaud A, de La Rochebrochard E, Alberti C (2020). Participatory interventions for sexual health promotion for adolescents and young adults on the internet: systematic review. J Med Internet Res.

[47] McKeon G, Papadopoulos E, Firth J, Joshi R, Teasdale S, Newby J, Rosenbaum S (2022). Social media interventions targeting exercise and diet behaviours in people with noncommunicable diseases (NCDs): a systematic review. Internet Interv.

[48] Mersha AG, Bryant J, Booth K, Watson L, Kennedy M (2024). The effectiveness of internet-based group behavioural interventions on lifestyle modifications: a systematic review. Prev Med.

[49] Niu Z, Bhurosy T, Heckman CJ (2022). Digital interventions for promoting sun protection and skin self-examination behaviors: a systematic review. Prev Med Rep.

[50] O'Connor H, Willcox JC, de Jersey S, Wright C, Wilkinson SA (2024). Digital preconception interventions targeting weight, diet and physical activity: a systematic review. Nutr Diet.

[51] Orchard LJ, Nicholls W (2020). A systematic review exploring the impact of social media on breastfeeding practices. Curr Psychol.

[52] Ou L, Chen AC, Amresh A (2023). The effectiveness of mHealth interventions targeting parents and youth in human papillomavirus vaccination: systematic review. JMIR Pediatr Parent.

[53] Seid A, Fufa DD, Bitew ZW (2024). The use of internet-based smartphone apps consistently improved consumers' healthy eating behaviors: a systematic review of randomized controlled trials. Front Digit Health.

[54] Sewak A, Yousef M, Deshpande S, Seydel T, Hashemi N (2023). The effectiveness of digital sexual health interventions for young adults: a systematic literature review (2010-2020). Health Promot Int.

[55] Talie Fenta E, Bogale EK, Anagaw TF (2024). The role of social media on COVID-19 preventive behaviors worldwide, systematic review. PLoS One.

[56] Watson-Mackie K, Arundell L, Lander N, McKay FH, Jerebine A, Venetsanou F, Barnett LM (2024). Technology-supported physical activity and its potential as a tool to promote young women's physical activity and physical literacy: systematic review. J Med Internet Res.

[57] Wu JJY, Ahmad N, Samuel M, Logan S, Mattar CNZ (2021). The influence of web-based tools on maternal and neonatal outcomes in pregnant adolescents or adolescent mothers: mixed methods systematic review. J Med Internet Res.

[58] Yeh JC, Niederdeppe J, Lewis NA, Jernigan DH (2023). Social media campaigns to influence alcohol consumption and related harms, attitudes, and awareness: a systematic review. J Stud Alcohol Drugs.

[59] An R, Ji M, Zhang S (2017). Effectiveness of social media-based interventions on weight-related behaviors and body weight status: review and meta-analysis. Am J Health Behav.

[60] Chau MM, Burgermaster M, Mamykina L (2018). The use of social media in nutrition interventions for adolescents and young adults-a systematic review. Int J Med Inform.

[61] Guse K, Levine D, Martins S, Lira A, Gaarde J, Westmorland W, Gilliam M (2012). Interventions using new digital media to improve adolescent sexual health: a systematic review. J Adolesc Health.

[62] Jones K, Eathington P, Baldwin K, Sipsma H (2014). The impact of health education transmitted via social media or text messaging on adolescent and young adult risky sexual behavior: a systematic review of the literature. Sex Transm Dis.

[63] Laranjo L, Arguel A, Neves AL, Gallagher AM, Kaplan R, Mortimer N, Mendes GA, Lau AYS (2015). The influence of social networking sites on health behavior change: a systematic review and meta-analysis. J Am Med Inform Assoc.

[64] Naslund JA, Kim SJ, Aschbrenner KA, McCulloch LJ, Brunette MF, Dallery J, Bartels SJ, Marsch LA (2017). Systematic review of social media interventions for smoking cessation. Addict Behav.

[65] Brijnath B, Protheroe J, Mahtani KR, Antoniades J (2016). Do web-based mental health literacy interventions improve the mental health literacy of adult consumers? Results from a systematic review. J Med Internet Res.

[66] Carson KV, Ameer F, Sayehmiri K, Hnin K, van Agteren JE, Sayehmiri F, Brinn MP, Esterman AJ, Chang AB, Smith BJ (2017). Mass media interventions for preventing smoking in young people. Cochrane Database Syst Rev.

[67] Hsu MSH, Rouf A, Allman-Farinelli M (2018). Effectiveness and behavioral mechanisms of social media interventions for positive nutrition behaviors in adolescents: A systematic review. J Adolesc Health.

[68] Maher CA, Lewis LK, Ferrar K, Marshall S, De Bourdeaudhuij I, Vandelanotte C (2014). Are health behavior change interventions that use online social networks effective? A systematic review. J Med Internet Res.

[69] Elaheebocus SMRA, Weal M, Morrison L, Yardley L (2018). Peer-based social media features in behavior change interventions: systematic review. J Med Internet Res.

[70] Williams G, Hamm MP, Shulhan J, Vandermeer B, Hartling L (2014). Social media interventions for diet and exercise behaviours: a systematic review and meta-analysis of randomised controlled trials. BMJ Open.

[71] Adepoju VA, Umebido C, Adelekan A, Onoja AJ (2023). Acceptability and strategies for enhancing uptake of human immunodeficiency virus self-testing in Nigeria. World J Methodol.

[72] Wu J, Benjamin EJ, Ross JC, Fetterman JL, Hong T (2025). Health messaging strategies for vaping prevention and cessation among youth and young adults: a systematic review. Health Commun.

[73] Martin C, MacDonald BH (2020). Using interpersonal communication strategies to encourage science conversations on social media. PLoS One.

[74] Kaplan A, Haenlein M (2014). Collaborative projects (social media application): about Wikipedia, the free encyclopedia. Business Horizons.

[75] Hamm MP, Shulhan J, Williams G, Milne A, Scott SD, Hartling L (2014). A systematic review of the use and effectiveness of social media in child health. BMC Pediatr.

[76] Shiyab W, Halcomb E, Rolls K, Ferguson C (2023). The impact of social media interventions on weight reduction and physical activity improvement among healthy adults: systematic review. J Med Internet Res.

[77] Aichner T, Grünfelder M, Maurer O, Jegeni D (2021). Twenty-five years of social media: a review of social media applications and definitions from 1994 to 2019. Cyberpsychol Behav Soc Netw.

[78] Lefebvre RC (2012). Transformative social marketing: co?creating the social marketing discipline and brand. J Soc Mark.

[79] Parackal M, Parackal S, Mather D, Eusebius S (2021). Dynamic transactional model: a framework for communicating public health messages via social media. Perspect Public Health.

[80] Akdeniz E, Borschewski KE, Breuer J, Voronin Y (2022). Sharing social media data: The role of past experiences, attitudes, norms, and perceived behavioral control. Front Big Data.

[81] Moullin JC, Dickson KS, Stadnick NA, Albers B, Nilsen P, Broder-Fingert S, Mukasa B, Aarons GA (2020). Ten recommendations for using implementation frameworks in research and practice. Implement Sci Commun.

[82] Kirk MA, Kelley C, Yankey N, Birken SA, Abadie B, Damschroder L (2016). A systematic review of the use of the consolidated framework for implementation research. Implement Sci.

[83] Nilsen P (2015). Making sense of implementation theories, models and frameworks. Implement Sci.

[84] Rogers T, Goldstein NJ, Fox CR (2018). Social mobilization. Annu Rev Psychol.

[85] Cardoso G, Lapa T, Di Fátima B (2016). People are the message. Social mobilization and social media in Brazil.

[86] Mehmet M, Roberts R, Nayeem T (2020). Using digital and social media for health promotion: a social marketing approach for addressing co-morbid physical and mental health. Aust J Rural Health.

[87] Shawky S, Kubacki K, Dietrich T, Weaven S (2019). Using social media to create engagement: a social marketing review. J Soc Mark.

