# Supplementary material for: Using Social Media to Disseminate Behavior Change Interventions: Scoping Review of Systematic Reviews
Source: J Med Internet Res. 2025 Jun 20;27:e57370. doi: 10.2196/57370 (PMC12228004; doi:10.2196/57370)
Supplement: Multimedia Appendix 2 [file jmir_v27i1e57370_app2.docx]

**Epistemonikos search strategy**

**11/07/2024 n=613**

(title:(("social media" OR facebook OR twitter OR whatsapp OR pinterest OR instagram OR tumblr OR flickr OR weibo OR ("social networking" AND (platform OR platforms OR websites OR website OR sites)))) OR abstract:(("social media" OR facebook OR twitter OR whatsapp OR pinterest OR instagram OR tumblr OR flickr OR weibo OR ("social networking" AND (platform OR platforms OR websites OR website OR sites)))))

AND

(title:((disseminate OR dissemination OR disseminating OR "scaling up" OR "scale up" OR spread OR spreading OR diffusion OR promote OR promoting OR target OR targeting OR engage OR engaging OR reach OR implement OR implementation OR translate OR translation OR translating OR "information-dissemination")) OR abstract:((disseminate OR dissemination OR disseminating OR "scaling up" OR "scale up" OR spread OR spreading OR diffusion OR promote OR promoting OR target OR targeting OR engage OR engaging OR reach OR implement OR implementation OR translate OR translation OR translating OR "information-dissemination”)))

AND

(title:(“behavior” OR “behaviour” OR “behavioral” OR “behavioural”) OR abstract:(“behavior” OR “behaviour” OR “behavioural))
