# Supplementary material for: Using Social Media to Disseminate Behavior Change Interventions: Scoping Review of Systematic Reviews
Source: J Med Internet Res. 2025 Jun 20;27:e57370. doi: 10.2196/57370 (PMC12228004; doi:10.2196/57370)
Supplement: Multimedia Appendix 3 [file jmir_v27i1e57370_app3.docx]

| Study | Number of included studies | Behavior change | Target population | Ingredients of the social media dissemination strategies | Outcomes | Theory |
| --- | --- | --- | --- | --- | --- | --- |
| Acuna 2020 [29] | 11 | Cancer preventive health behaviors (i.e., sunscreen use, smoking, and diet) and/or early detection strategies (i.e., screening testing and/or self-skin exams) | General population | One-way spread: watching videos on YouTube | Impact on health and health-related behaviors.  User Perception: knowledge and intention, Information preference | Not Mentioned |
| Al-Dhahir 2022  [30] | 59 | Lifestyle modification (weight loss, physical activity, healthy eating, smoking, and alcohol use) for people with a low SES | General population | Not enough information provided | Impact on health and health-related behaviors.  User Perception | Social cognitive theory, I-Change Model, Theory of Planned Behavior, Health Belief Model, theories of self-regulation, Precaution Adoption Process Model |
| An 2017  [59] | 27 | Physical activity and weight management | Patients: adults living with obesity, metabolic syndrome, and cancer survivors | One-way spread on Instagram, Facebook, Twitter | Impact on health and health-related behaviors: BMI, weight measures, and body fat | Social Cognitive Theory |
| Bhatt 2021 [31] | 5 | Adoption of clinical practice guidelines | Multiple groups: Clinicians, health workers, medical students, patients | One-way spread on Twitter, YouTube, LinkedIn, Facebook  Invoking conversations: discussion board, Twitter chat | Diffusion and reach  Impact on health and health-related behaviors: adherence  User perception | Not Mentioned |
| Brigden 2020 [32] | 17 | Behavior change in chronic health conditions (e~~.~~g~~.~~, obesity, anxiety, ADHD, Asthma, Cerebral Palsy) | Children aged between 5 and 12 years. | One-way spread on web-based platforms  Invoking conversations: discussion on WhatsApp | Impact on health and health-related behaviors. | Not Mentioned |
| Brijnath 2016 [65] | 14 | Literacy and behavior related to mental illness | Adult participants with a clinical indication of a mental illness | Miscellaneous: Facebook game | Impact on health and health-related behaviors  User Perception: Perceived stigma about mental illness | Not Mentioned |
| Buja 2024 [33] | 19 | Physical activity | University Students | One-way spread on Facebook  Peer motivation to increase steps/day in a Facebook group. | Impact on health and health-related behaviors.  User Perception (Levels of Perceived Stress).  Social support for physical activity | Social Cognitive Theory, Cognitive Behavior Framework, and Self-Determination Theory |
| Carson 2017 [66] | 30 | Smoking Cessation | Young people under the age of 25 years | One-way spread on Facebook, YouTube, and Twitter | Impact on health and health-related behaviors.  User Perception | Social Cognitive Theory, Social Learning Theory |
| Chau 2018 [60] | 16 | Maintaining a healthy diet | Adolescents (10-19) and/or young adults (18-25) who are healthy, or have a health challenge, such as being overweight, living with obesity, or having a chronic disease | One-way spread on Twitter, Facebook  Invoking conversations: Facebook, WhatsApp, Twitter chat | Impact on health and health-related behaviors  Diffusion and reach of app and website usage, frequency of interactions, reading/viewing content, and participation in peer communication activities.  User satisfaction | Theory of Reasoned Action, Self-Determination Theory, Trans-contextual model of motivation, Theory of Planned Behavior, Attitude-Social Influence-Self-efficacy model based on the Theory of Planned Behavior, Social Cognitive Theory, social network theories |
| Choi 2023 [34] | 24 | HPV vaccination | Adolescents and their parents | One-way spread on Facebook  Invoking conversations: Facebook  Miscellaneous: game-based interventions | Impact on health and health-related behaviors. | Information systems research frameworks, Health Belief Model, Inoculation Theory, Social Cognitive Theory, Theory of Planned Behavior, Theory of Reasoned Action, IDM (Informed Decision Making), Diffusion of Innovations Theory, Transportation Theory, Transtheoretical Model, Integrated Behavioral Model, Intervention Mapping, Self-Determination Theory, and Theoretical Domains Framework. |
| de Oliveira Júnior 2023 [35] | 12 | Oral health | General public | One-way spread on Facebook | Impact on health and health-related behaviors.  User Perception | Not Mentioned |
| Draganidis 2024 [36] | 14 | Help seeking in mental illness | General public | One-way spread on Twitter, YouTube, Instagram, Facebook  Invoking conversations: Facebook, Twitter chat | Impact on health and health-related behaviors  Engagement  User Perception: | Health Belief Model, Transtheoretical Model of Behavior Change. |
| Elaheebocus 2018 [69] | 143 | Healthy diet and physical activity | General public | One-way spread on Twitter, YouTube, Facebook  Invoking conversations: Facebook, Twitter chat  Peer Motivation: using a private group chat | Impact on health and health-related behaviors | Theory of Planned Behavior, Social cognitive Theory, Cognitive behavioral approach, Principles of web usability, Transtheoretical Model of Change, theory of social support, Social Learning Theory,  cognitive–behavioral and self-control principles, Goal Setting Theory,  Social Comparison,  Self-Determination Theory |
| Eppes 2023 [37] | 23 | Healthy diet, physical activity, and breastfeeding practices | Vulnerable families | One-way spread on Twitter, Facebook  Invoking conversations: Facebook, Twitter chat  Peer Motivation: using a private group chat | Engagement  Impact on health and health-related behaviors | Social cognitive theory, Health Belief Model, Transtheoretical Model, Cognitive Social Health Information Processing, Health Action Process Approach, Socioecological model, Theory of Planned Behavior |
| Goodyear 2021 [38] | 16 | Healthy diet and physical activity | Young people and adults | One-way spread on Twitter, Facebook  Invoking conversations: Facebook, Twitter chat | Impact on health and health-related behaviors engagement | Not mentioned |
| Guse 2012 [61] | 10 | Sexual health | Adolescents | One-way spread on MySpace  Invoking conversations: Bulletin board | Impact on health and health-related behaviors: sexual behavior, Removal of MySpace sex references, Removal of MySpace substance abuse references, MySpace profile security set to private.  User Perception: Perceived importance and self-efficacy regarding abstinence and condom use, knowledge about EC and abortion law, permissiveness toward premarital sex and contraceptive use | Social Cognitive Theory, Theory of Planned Behavior, Theory of Reasoned Action, the extended parallel process model, Transtheoretical Model |
| Hsu 2018 [67] | 14 | Healthy diet | General population: Adolescents aged 13-18 years | One-way spread on blogs  Invoking conversations: discussion boards and forums | Impact on health and health-related behaviors: adherence  User Perception: Perceived behavioral control | Social Cognitive Theory, Mentoring models, ASE model: attitude, social influence, self-efficacy, Theory of Interactive Technology, Social Learning Theory, Health Promotion reference model |
| Ibrahim 2024 [39] | 23 | HIV/AIDS prevention and control | General public: Men who have sex with men, people living with HIV, adolescents, and college students | One-way spread on Twitter, Instagram, Facebook, and LinkedIn  Invoking conversations: Facebook groups | Impact on health and health-related behaviors: HIV testing, behavioral skills social support for HIV test and also adherence to HIV treatment  User Perception: condom use attitudes | Not mentioned |
| Jones 2014 [62] | 11 | Sexual health | General population: adolescents and young adults, aged 13-29 years | One-way spread on Facebook | User perception  Impact on health and health-related behaviors: sexual health behavior | Not mentioned |
| Kim 2024 [40] | 25 | Hepatitis B testing | General population | One-way spread on WeChat | Impact on health and health-related behaviors: HBV surface antigen (HBsAg) and HCV antibody (anti-HCV IgG) test uptake | Not mentioned |
| Kudrati 2022 [41] | 8 | Pre-exposure prophylaxis | Black and Latinx MSM and women under the age of 29 years | One-way spread on Facebook and Instagram  Peer Motivation: using a Facebook group | Impact on health and health-related behaviors: PrEP uptake  User Perception: Pre-exposure prophylaxis (PrEP) awareness | Social Cognitive Theory |
| Kulandaivelu 2023 [42] | 28 | Healthy dietary behavior | Adolescents | One-way spread on Facebook  Invoking conversations: Facebook groups | Impact on health and health-related behaviors: Improvements in BMI and dietary behavior | Theory of Interactive Technology, Social Learning Theory, Cognitive behavioral approaches, Social Cognitive Theory, community mobilization, and social network theory, Reader-to-Leader Framework, Technology Acceptance Model, Transtheoretical Model |
| Kuwahara 2023 [43] | 7 | COVID-19 preventive behaviors, vaccination, or information-seeking behaviors | General Public | One-way spread on Facebook | Impact on health and health-related behaviors: COVID-19 infection, preventive behaviors, such as vaccination uptake, information-seeking | Not mentioned |
| Laranjo 2015 [63] | 12 | fitness, sexual health, food safety, smoking, and health promotion | General public | One-way spread on Facebook  Peer Motivation: using a Facebook group | Impact on health and health-related behaviors: physical activity change, dietary habits, smoking cessation, alcohol consumption, and sexual behaviors | Social network theory and Social Cognitive Theory |
| Li 2021 [44] | 20 | COVID-19 vaccine uptake | General Public | One-way spread on Twitter, Facebook, and YouTube  Invoking conversations: Facebook and WhatsApp | Impact on health and health-related behaviors: increase in vaccine doses, Knowledge of specific diseases and their vaccination.  Engagement through assessment of the number of likes, comments, and shares  User Perception: improvements in attitudes regarding vaccination benefits, | Health Belief Model, Theory of Planned Behavior, and Social Cognitive Theory |
| Limaye 2021 [45] | 46 | Vaccine uptake | General public | One-way spread on Twitter, Facebook, and Instagram  Invoking conversations: Facebook and WhatsApp | Impact on health and health-related behaviors: Vaccine knowledge, intentions/ behaviors.  Engagement through assessment of the number of likes, comments, shares, and tweets  User Perception: Vaccination attitude | Not mentioned |
| Maher 2014 [68] | 10 | Key modifiable health behaviors (tobacco and alcohol consumption, dietary intake, physical activity, and sedentary behavior) | General public | One-way spread on Twitter, Facebook, and Podcasts  Invoking conversations: discussion board and forum, Facebook | Impact on health and health-related behaviors: BMI, physical activity, smoking | Social Cognitive Theory, Social Learning Theory, Theory of Planned Behavior |
| Martin 2020 [46] | 60 | Sexual health | Adolescents and young adults | Invoking conversations: Discussion forums and Facebook  Peer motivation: Facebook group chat  Miscellaneous: Online games | Impact on health and health-related behaviors: Behaviors, knowledge, communication, skills, self-efficacy, use of contraception, incidence, or history of STIs, HIV stigma and test history, homophobia, partner violence  User perception: Acceptability, attractiveness, feasibility, satisfaction, and implementation of the intervention process. | Information-Motivation-Behavioral skills model; Social Identity Theory; Social Cognitive Theory; Social Learning Theory |
| McKeon 2022 [47] | 8 | Physical activity and healthy eating | General Public | One-way spread on Facebook  Invoking conversations: online discussion boards, online bulletin boards, chat rooms, online community, and Facebook  Peer motivation: Facebook group chat | Impact on health and health-related behaviors: minutes of physical activity, sedentary time, step count or changes in overall food consumption, nutrient, mineral, and/or supplement intake  User perception: feasibility, acceptability, safety defined by adverse events, usability, | Social Cognitive Theory, Transtheoretical Model, Self-efficacy theory, Theory of Self-regulation |
| Mersha 2024 [48] | 32 | smoking, nutrition, alcohol consumption, physical activity, and obesity | Adults | One-way spread on Facebook  Invoking conversations: online discussion boards, online bulletin boards, chat rooms, online community, Twitter, and Facebook | Impact on health and health-related behaviors: Smoking, Alcohol, Nutrition, Physical activity, Obesity | Not mentioned |
| Naslund 2017 [64] | 7 | Smoking cessation | General Public | One-way spread on Facebook  Invoking conversations: Twitter, WhatsApp, Facebook  Peer motivation: Facebook group chat  Miscellaneous: Facebook-based game | User perception: feasibility, acceptability, safety, usability, efficacy, implementation, or effectiveness.  Engagement through posting comments or liking content.  Impact on health and health-related behaviors: abstinence, the number of relapses, and the number of quit attempts. | Not mentioned |
| Niu 2022 [49] | 62 | Promoting sun protection and skin self-examination | General Public | One-way spread on Facebook  Invoking conversations: Twitter, WhatsApp, Facebook | Impact on health and health-related behaviors: sun protection or ultraviolet radiation (UV) exposure-related outcomes.  User Perception: cognitive outcomes, such as assessed knowledge of skin cancer or melanoma, the perceived risk of getting skin cancer. | Not mentioned |
| O'Connor 2023 [50] | 10 | Physical activity and healthy eating | Women of childbearing age, older than 18 years, who were not currently pregnant and were between pregnancies or actively trying to conceive | One-way spread on Facebook | Impact on health and health-related behaviors: changes in women's body weight, BMI  Engagement through posting comments or likes. | Not mentioned |
| Orchard 2020 [51] | 22 | Breast-feeding practices | Breastfeeding mothers, pregnant mothers, parents, and expecting or prospective parents. | One-way spread on Facebook and Twitter  Invoking conversations: Facebook  Peer motivation: Facebook group chat | Impact on health and health-related behaviors: duration of breastfeeding  User Perception: Breastfeeding, Intention, Attitude towards Breastfeeding, and Breastfeeding Self-Efficacy.  Engagement: page popularity (high or low, as measured through page likes, shares, and comment likes) | Not mentioned |
| Ou 2023  [52] | 17 | HPV vaccination | Unvaccinated youth and their parents | One-way spread on Facebook and Twitter  Invoking conversations: Facebook | Engagement: clicks, likes, comments, and shares.  User Perception: HPV-Associated Knowledge, Attitudes, and Beliefs  Impact on health and health-related behaviors: Vaccine uptake and completion | Not mentioned |
| Seid 2024 [53] | 39 | Healthy eating | Healthy adults,  overweight or living with obesity, chronically ill patients, pregnant mothers, and children of different age groups. | Not enough information provided | Impact on health and health-related behaviors: nutrition, breastfeeding, reading nutrition labels, and preventing food contamination. | Not mentioned |
| Sewak 2023 [54] | 61 | Sexual health | Young adults and adolescents | One-way spread on Facebook  Invoking conversations: Facebook chat  Peer Motivation: using a Facebook group chat | Impact on health and health-related behaviors: promoting sexual health-related behaviors, promoting sexual health-related products and services, and impact (viral load).  User perception | Not mentioned |
| Simeon 2020 [16] | 71 | HIV testing, mental health, physical activity, smoking cessation, and vaccination | Adults | Peer Motivation: using Facebook, Twitter, and WhatsApp | Impact on health and health-related behaviors: health-related behaviors, physical health, well-being, and psychological health. | Not mentioned |
| Talie 2024 [55] | 32 | COVID-19 preventive behaviors | General Public | One-way spread on Facebook, WhatsApp, LinkedIn, and YouTube  Invoking conversations: group chat | Engagement by expressing likes, by writing comments, or by sharing its contents.  Impact on health and health-related behaviors: Precautionary behaviors about COVID-19 (such as hand hygiene, mask-wearing, and social distancing)  User perception: fear or concern about COVID-19 | Not mentioned |
| Williams 2014 [70] | 22 | Diet and exercise | Overweight individuals, healthy sedentary individuals | One-way spread on Twitter, Instagram, blogs  Invoking conversations: Discussion boards and forums, bulletin boards  Peer motivation: Posting progress on Facebook by users | Impact on health and health-related behaviors: BMI, weight measures, body fat, daily activities | Not mentioned |
| Watson-Mackie 2024 [56] | 23 | Physical activity | Young women | Not enough information provided | Impact on health and health-related behaviors: self-reported physical activity | Social Cognitive Theory, Transtheoretical Model of Behavior Change, and Self-Determination Theory |
| Wu 2021 [57] | 7 | Vaping cessation | Adolescent mothers and pregnant adolescents; first-time mothers aged 18-21 | One-way spread on Facebook  Invoking conversations: private Facebook group, Discussion boards and forums, bulletin board | Impact on health and health-related behaviors: Maternal: mental health, parental outcome, pregnancy complications, birth preparedness, Infant: Healthcare use, breastfeeding, birth weight, gestational age, child protection  User Perception: Attitudes and Beliefs about Prenatal Health. | Not mentioned |
| Yeh 2023 [58] | 11 | Alcohol consumption | General Public | One-way spread on Facebook | Impact on health and health-related behaviors: Self-reported drunk driving and related behaviors; motor vehicle crash data; behaviors; Self-reported drinking  beliefs and behaviors; campaign awareness  User Perception: awareness of designated driver program, Exposure and opinion toward media campaign, drinking | Social norms theory, Social Cognitive Theory, Theory of Planned Behavior, Bystander education model, social marketing, Value network, holistic marketing |
